# The genome sequence of the eastern grey squirrel, *Sciurus carolinensis* Gmelin, 1788

**DOI:** 10.12688/wellcomeopenres.15721.1

**Published:** 2020-02-13

**Authors:** Dan Mead, Kathryn Fingland, Rachel Cripps, Roberto Portela Miguez, Michelle Smith, Craig Corton, Karen Oliver, Jason Skelton, Emma Betteridge, Jale Doulcan, Michael A. Quail, Shane A. McCarthy, Kerstin Howe, Ying Sims, James Torrance, Alan Tracey, Richard Challis, Richard Durbin, Mark Blaxter

**Affiliations:** 1 Tree of Life, Wellcome Sanger Institute,Wellcome Genome Campus, Hinxton, CB10 1SA, UK; 2 Nottingham Trent University, School of Animal, Rural and Environmental Sciences, Nottingham, NG25 0QF, UK; 3 Red Squirrel Officer, The Wildlife Trust for Lancashire, Manchester and North Merseyside, The Barn, Berkeley Drive, Bamber Bridge, Preston, PR5 6BY, UK; 4 Department of Life Sciences, Natural History Museum, London, SW7 5BD, UK

**Keywords:** Sciurus carolinensis, grey squirrel, genome sequence, chromosomal

## Abstract

We present a genome assembly from an individual male *Sciurus carolinensis* (the eastern grey squirrel; Vertebrata; Mammalia; Eutheria; Rodentia; Sciuridae). The genome sequence is 2.82 gigabases in span. The majority of the assembly (92.3%) is scaffolded into 21 chromosomal-level scaffolds, with both X and Y sex chromosomes assembled.

## Species taxonomy

Eukaryota; Metazoa; Chordata; Craniata; Vertebrata; Euteleostomi; Mammalia; Eutheria; Euarchontoglires; Glires; Rodentia; Sciuromorpha; Sciuridae; Sciurinae; Sciurini; Sciurus; *Sciurus carolinensis* Gmelin, 1788 (NCBI txid 30640).

## Background

The eastern grey squirrel, *Sciurus carolinensis*, is native to eastern North America, where it plays important roles in forest regeneration through its habit of caching food nuts and seeds ( Corbet & Hill, 1991) ^1^. In North America, *S. carolinensis* has been introduced outside its native range such that it is now found from the Canadian Pacific northwest to Florida. *S. carolinensis* was introduced to Britain (in 1876), Ireland (in 1911), Italy (in 1948), South Africa (before 1900), Australia (in 1880s, extirpated in 1973) and Pitcairn island (in 1987) (see https://www.cabi.org/isc/datasheet/49075). *S. carolinensis*, which thrives in urban parklands and gardens, is classed as invasive in Europe and on Pitcairn island. In Britain and Ireland the expansion of *S. carolinensis* populations has driven decline in populations of the native red squirrel, *Sciurus vulgaris*, which we have also assembled ( Mead *et al.,* 2020). The negative impact of *S. carolinensis* is through interspecific competition, leading to competitive exclusion of *S. vulgaris*, and by their carriage of squirrelpox virus, to which they are resistant but *S. vulgaris* are not ( Chantrey *et al.,* 2014) ( Darby *et al.,* 2014). The *S. carolinensis* genome will aid analyses of resistance and susceptibility to squirrelpox, as well as to the genomics of invasiveness.

## Genome sequence report

**Figure 1.**
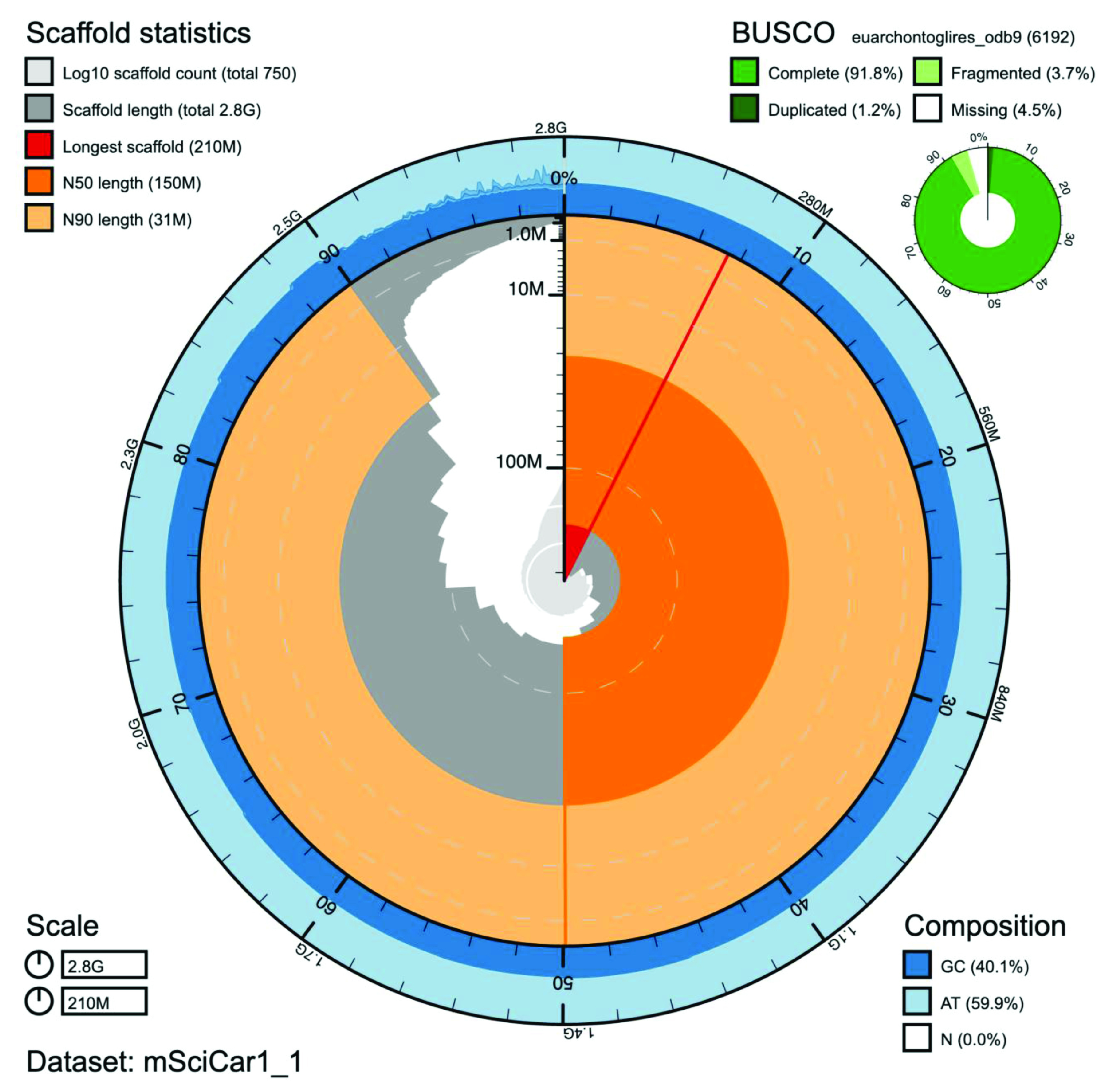
Genome assembly of *Sciurus carolinensis* mSciCar1: Metrics. BlobToolKit Snailplot showing N50 metrics for *S. carolinensis* assembly mSciCar1 and BUSCO scores for the Euarchontoglires set of orthologues. The interactive version is available here.

The genome was sequenced from DNA extracted from a naturally deceased male *S. carolinensis* collected as part of a squirrel monitoring project run by the Wildlife Trust for Lancashire, Manchester and North Merseyside. A total of 74-fold coverage in Pacific Biosciences single-molecule long reads (N50 28 kb) and 40-fold coverage in 10X Genomics read clouds (from molecules with an estimated N50 of 19 kb) were generated. Primary assembly contigs were scaffolded with chromosome conformation HiC data (42-fold coverage). A contamination check identified a small number of low-coverage contigs that were likely to have derived from an apicomplexan parasite infecting the squirrel ( Léveillé *et al*., 2020); these were removed. Subsequent manual assembly curation corrected 272 missing/misjoins and removed three haplotypic duplications, reducing the scaffold number by 19% and increasing the scaffold N50 by 242% The final assembly has a total length of 2.82 Gb in 752 sequence scaffolds with a scaffold N50 of 148.2 Mb ( Table 1). The majority, 92.3%, of the assembly sequence was assigned to 21 chromosomal-level scaffolds representing 19 autosomes (numbered by sequence length), and the X and Y sex chromosomes ( Figure 1– Figure 5; Table 2) plus 13 unlocalised scaffolds (assigned to chromosomes but with ambiguous placement). The assembly has a BUSCO ( Simão *et al.,* 2015) completeness of 93.7% using the mammalia_odb9 reference set. The primary assembly is a large-scale mosaic of both haplotypes (i.e. is not fully phased) and we have therefore also deposited the contigs corresponding to the alternate haplotype. The *S. carolinensis* mSciCar1 genome sequence is largely collinear with that of *S. vulgaris* mSciVul1 ( Figure 4).

**Table 1.** Genome data for Sciurus carolinensis mSciCar1.

Project accession data	
Assembly identifier	mSciCar1
Species	*Sciurus carolinensis*
Specimen	NHMUK ZD 2019.214
NCBI taxonomy ID	30640
BioProject	PRJEB35386
Biosample ID	SAMEA994726
Isolate information	Wild isolate; male
Raw data accessions	
PacificBiosciences SEQUEL I	ERR3313242-ERR3313245, ERR3313247-ERR3313255, ERR3313329, ERR3313331, ERR3313332, ERR3313342- ERR3313348
10X Genomics Illumina	ERR3316153-ERR3316156, ERR3316173-ERR3316176
Hi-C Illumina	ERR3312499-ERR3312500, ERR3850937
Genome assembly	
Assembly accession	GCA_902686445.1
Accession of alternate haplotype	GCA_902685475.1
Span (Mb)	2,815,397,268
Number of contigs	2576
Contig N50 length (Mb)	13.98
Number of scaffolds	752
Scaffold N50 length (Mb)	148.23
Longest scaffold (Mb)	208.99
BUSCO * genome score	C:93.7%[S:92.3%,D:1.4%],F:2.8%,M :3.5%,n:4104

* BUSCO scores based on the mammalia_odb9 BUSCO set using v3.0.2. C= complete [S= single copy, D=duplicated], F=fragmented, M=missing, n=number of orthologues in comparison. A full set of BUSCO scores is available at https://blobtoolkit.genomehubs.org/view/mSciCar1_1/dataset/mSciCar1_1/busco.

**Figure 2.**
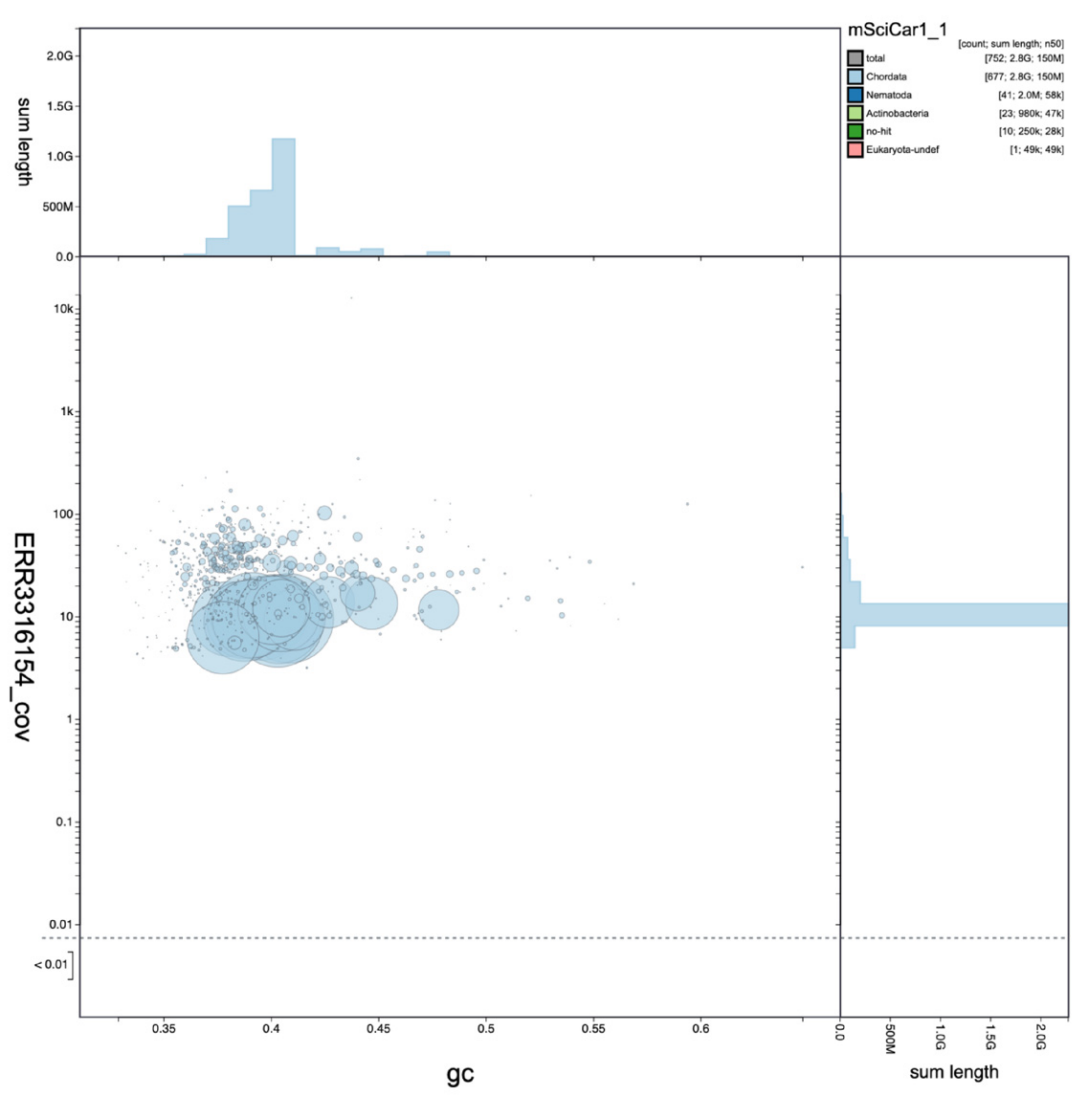
Genome assembly of Sciurus carolinensis mSciCar1: GC-coverage plot. BlobToolKit GC-coverage plot of *S. carolinensis* mSciCar1 from long read data submission ERR3316154. The interactive version is available here.

**Figure 3.**
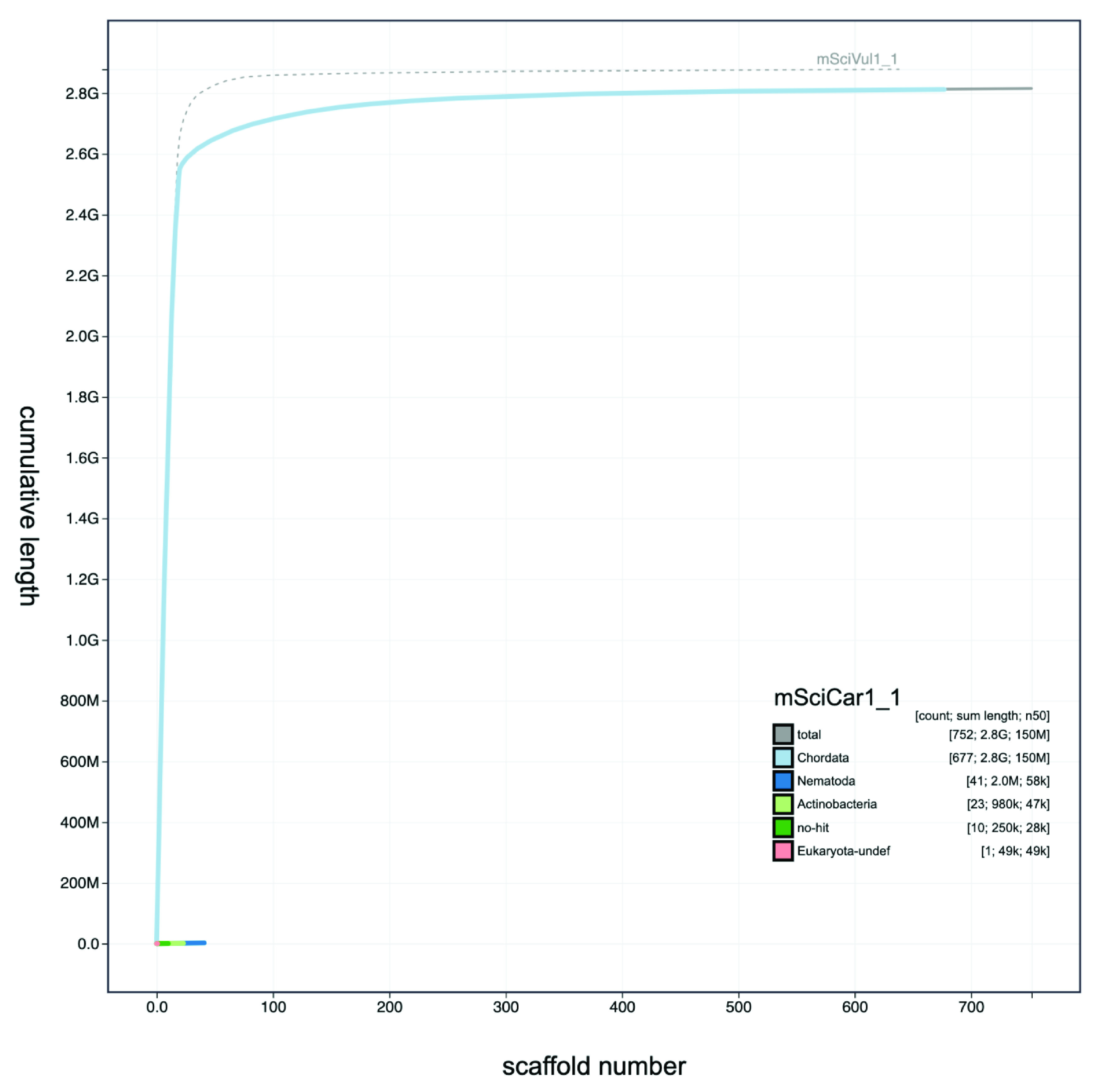
Genome assembly of *Sciurus carolinensis* mSciCar1: Cumulative sequence plot. The blue line in the main plot shows the cumulative sequence plot for mSciCar. The sashed line shows the cumulative sequence plot of *S. vulgaris* mSciVul1 for comparison. The interactive version is available here.

**Figure 4.**
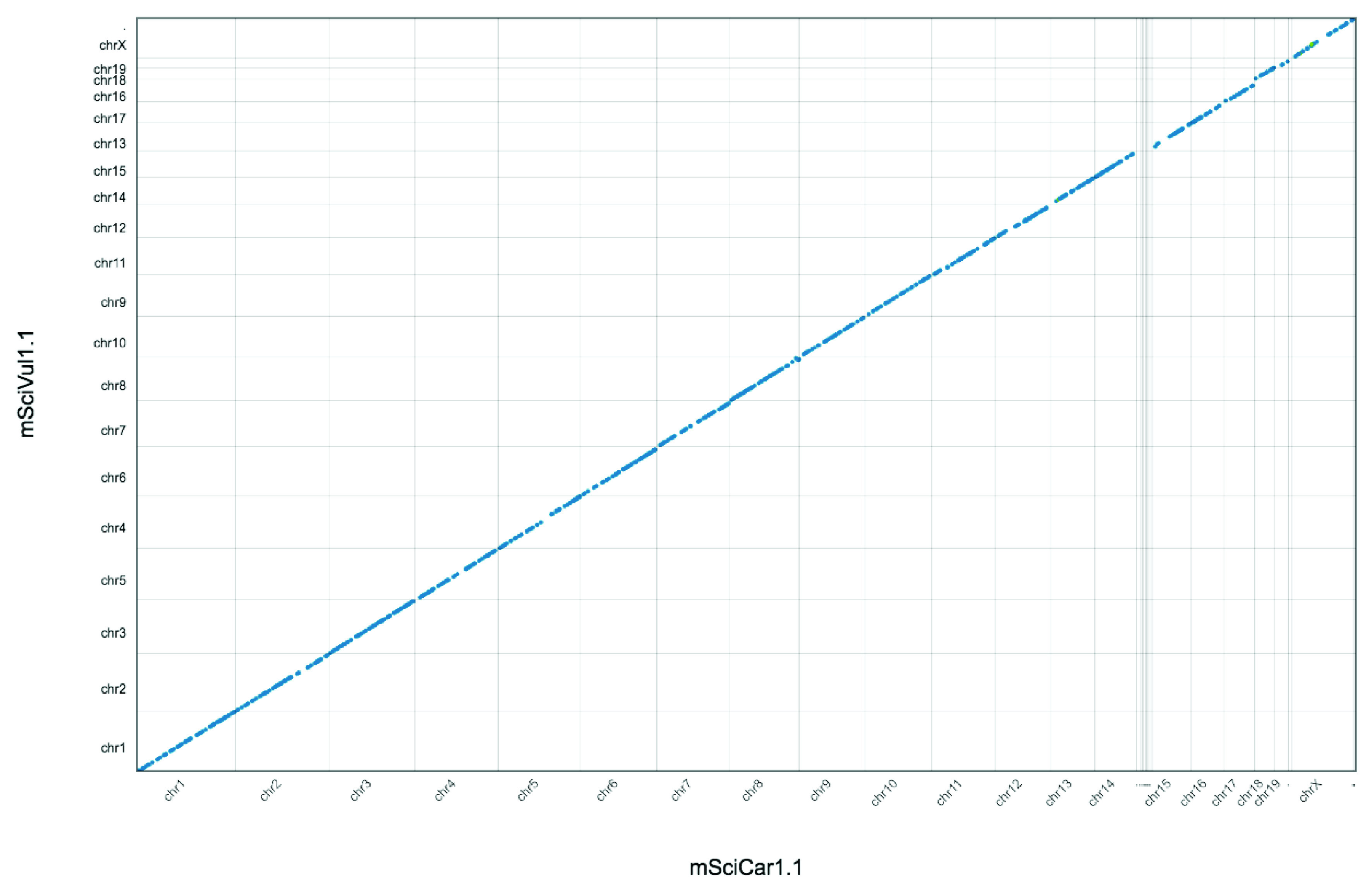
Genome assembly of *Sciurus carolinensis* mSciCar1: Whole genome alignment with *Sciurus vulgaris* mSciVul1. A nucmer ( Kurtz *et al.,* 2004) pairwise alignment of mSciCar1 (x-axis) with mSciVul1 (Y axis).

**Figure 5.**
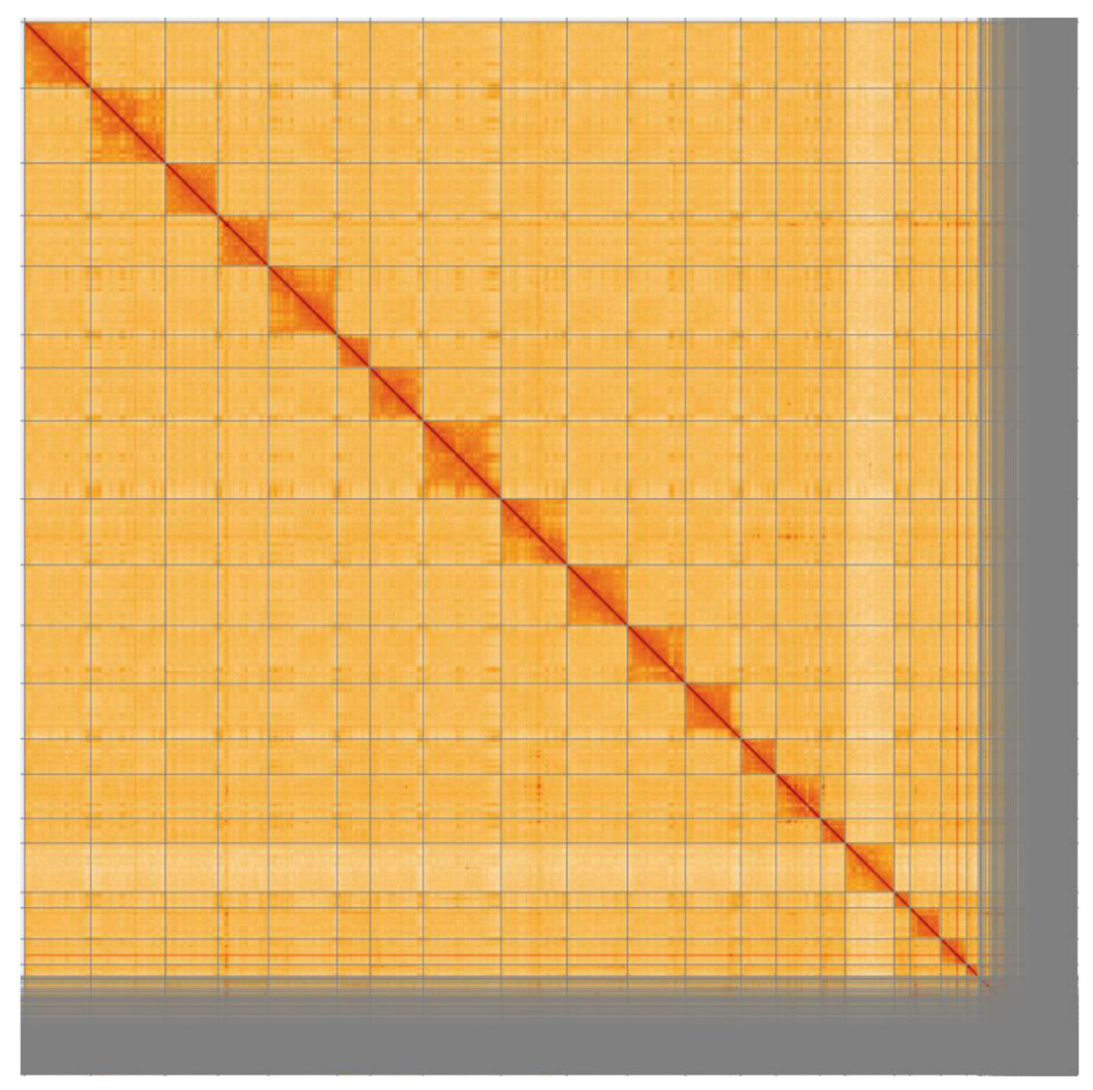
Genome assembly of *Sciurus carolinensis* mSciCar1: Hi-C contact map. Hi-C scaffolding of the *S. carolinensis* mSciCar1 assembly visualised in HiGlass ( Kerpedjiev *et al.,* 2018).

**Table 2.** Chromosomal pseudomolecules in the genome assembly of *Sciurus carolinensis* mSciCar1.

ENA accession	Chromosome	Size (Mb)	GC%
LR738590.1	1	208.99	40.3
LR738591.1	2	199.83	40.8
LR738592.1	3	183.55	40.3
LR738593.1	4	177.11	39.5
LR738594.1	5	175.91	39.1
LR738595.1	6	162.27	38.7
LR738596.1	7	154.99	39.1
LR738597.1	8	148.23	40.5
LR738598.1	9	141.42	38.8
LR738599.1	10	140.98	38.1
LR738600.1	11	135.23	40.1
LR738602.1	12	118.65	40.1
LR738603.1	13	94.68	41.1
LR738604.1	14	88.65	40.2
LR738605.1	15	83.14	40.5
LR738606.1	16	68.57	44.7
LR738607.1	17	66.05	42.7
LR738608.1	18	41.56	47.8
LR738609.1	19	30.99	44
LR738601.1	X	131.72	37.8
LR738610.1	Y	4.81	38.3
-	Unplaced	258.08	40

## Methods

The eastern grey squirrel specimen was collected by the Wildlife Trust for Lancashire, Manchester and North Merseyside as part of an ongoing programme of recovery of dead squirrels. A full tissue dissection and preservation in 80% ethanol was undertaken and the specimen accessioned by the Natural History Museum, London.

DNA was extracted using an agarose plug extraction from spleen tissue following the Bionano Prep Animal Tissue DNA Isolation Soft Tissue Protocol ^2^. Pacific Biosciences CLR long read and 10X Genomics read cloud sequencing libraries were constructed according to the manufacturers’ instructions. Sequencing was performed by the Scientific Operations core at the Wellcome Sanger Institute on Pacific Biosciences SEQUEL I (single molecule long read) and Illumina HiSeq X (10X Genomics Chromium). HiC data were generated using the Dovetail v1.0 kit and sequenced on HiSeq X.

See Table 3 for software versions and sources. Assembly was carried out using Falcon-unzip ( Chin *et al.,* 2016), haplotypic duplication was identified and removed with purge_dups ( Guan *et al.,* 2020) and a first round of scaffolding carried out with 10X Genomics read clouds using scaff10x. Scaffolding with Hi-C data was carried out using SALSA2. The Hi-C scaffolded assembly was polished with arrow using the PacBio data, then polished with the 10X Genomics Illumina data by aligning to the assembly with longranger align, calling variants with freebayes ( Garrison & Marth, 2012) and applying homozygous non-reference edits using bcftools consensus. Two rounds of the Illumina polishing were applied. The assembly was checked for contamination and corrected using the gEVAL system ( Chow *et al.,* 2016). Since Hi-C data were sparse, curation was aided by synteny with the assembly for *Sciurus vulgaris* simultaneously being curated by the Wellcome Sanger Institute. The genome was analysed within the BlobToolKit environment ( Challis *et al*., 2019).

**Table 3.** Software tools used.

Software tool	Version	Source
Falcon-unzip	falcon-kit 1.2.2	( Chin *et al*., 2016)
purge_dups	1.0.0	( Guan *et al*., 2020)
SALSA2	2.2	( Ghurye *et al*., 2018)
scaff10x	4.2	https://github.com/wtsi- hpag/Scaff10X
arrow	GenomicConsensus 2.3.3	https://github.com/ PacificBiosciences/ GenomicConsensus
longranger align	2.2.2	https:// support.10xgenomics. com/genome-exome/ software/pipelines/latest/ advanced/other-pipelines
freebayes	v1.1.0-3-g961e5f3	( Garrison & Marth, 2012)
bcftools consensus	1.9	http://samtools.github. io/bcftools/bcftools.html
gEVAL	2016	( Chow *et al*., 2016)
BlobToolKit	1	( Challis *et al*., 2019)
nucmer from MUMmer 3	3.0	( Kurtz *et al*., 2004)

## Data availability

### Underlying data

European Nucleotide Archive: *Sciurus carolinensis* (grey squirrel) genome assembly, mSciCar1. BioProject accession number PRJEB35386; https://identifiers.org/ena.embl:PRJEB35386.

The genome sequence is released openly for reuse. The *S. carolinensis* genome sequencing initiative is part of the Wellcome Sanger Institute’s “25 genomes for 25 years” project ^3^. It is also part of the Vertebrate Genome Project (VGP) ^4^ and the Darwin Tree of Life (DToL) project ^5^. The specimen has been preserved in ethanol and deposited with the Natural History Museum, London under registration number NHMUK ZD 2019.214, where it will remain accessible to the research community for posterity. All raw data and the assembly have been deposited in the ENA. The genome will be annotated and presented through the Ensembl pipeline at the European Bioinformatics Institute. Raw data and assembly accession identifiers are reported in Table 1.
