# Supplementary material for: The genome sequence of the eastern grey squirrel, Sciurus carolinensis Gmelin, 1788
Source: Wellcome Open Res. 2020 Feb 13;5:27. [Version 1] doi: 10.12688/wellcomeopenres.15721.1 (PMC7653645; doi:10.12688/wellcomeopenres.15721.1)
Supplement: Peer review history

# Peer review: Reviewer response for version 1

**DOI:** 10.21956/wellcomeopenres.17234.r40853

**Published:** 2020-11-09

**Authors:** Takafumi Katsumura

**Affiliations:** 1 Department of Anatomy, Kitasato University School of Medicine, Sagamihara, Japan

The authors constructed a whole-genome sequence for the eastern gray squirrel using spleen-derived DNA from naturally dead squirrels collected in a Wildlife Trust project. This genome sequence is based on long reads (74-fold coverage) from the PacBio sequencer, short reads from 10X Genomics + Illumina sequencer (40-fold coverage), and scaffolding by Hi-C data (42-fold coverage). From the various genome statistics calculated and BUSCO's score, I think this whole-genome sequence is useful for comparative genome studies.

 My comments are as follows:

1. I would suggest that the authors describe the age of the squirrel used as samples (if the authors know) and the collecting date. This information may be useful for future secondary use of the Hi-C data acquired in this paper. Also, I would suggest that the authors describe how the amount was the spleen used for DNA extraction.
2. I would suggest that the authors add the number of libraries used to generate the reads by the PacBio and Illumina in the text, respectively. The readers could know their numbers by looking and counting them in Table 1, but I think it will help the reader understand this work's data quality if specified in the text.
3. For the contact map in Figure 5, the authors should describe the X and Y axes and label them. Also, I think it would be easier for the reader to understand the figure if there is a color key.
4. To drive the reproducibility of the data by readers, it would be important to describe the settings of the software tools used to construct the genome sequence. I would suggest that the authors describe the settings of all software tools in the text.

Are sufficient details of methods and materials provided to allow replication by others?

Partly

Is the rationale for creating the dataset(s) clearly described?

Yes

Are the datasets clearly presented in a useable and accessible format?

Yes

Are the protocols appropriate and is the work technically sound?

Yes

Reviewer Expertise:

Evolutionary biology.

I confirm that I have read this submission and believe that I have an appropriate level of expertise to confirm that it is of an acceptable scientific standard.

# Peer review: Reviewer response for version 1

**DOI:** 10.21956/wellcomeopenres.17234.r37915

**Published:** 2020-02-17

**Authors:** Erik Garrison

**Affiliations:** 1 UC Santa Cruz Genomics Institute, University of California, Santa Cruz, Santa Cruz, CA, USA

The authors present the assembly of a male eastern grey squirrel, based on PacBio single molecule sequencing and 10X genomics linked read sequencing. The approach is technically very sound, and mirrors that used in the Vertebrate Genomes Project. The assembly's completeness is impressive, as the authors show by comparison to an existing assembly from the red squirrel, evaluation of the assembly against BUSCO, and several plots from BlobToolKit. I am confident that this assembly will be usable by researchers working on this species.

 I would suggest that the authors improve the rendering of several of the figures. Those produced by the BlobToolKit have very small font relative to their rasterized pixel density. I would either render them as vector graphics or adjust the rendering (if possible) to improve the font size. The HiGlass plot clearly demonstrates the expected pattern of connectivity across the chromosome-scale scaffolds, but overplotting of the delimiting line (grey bars) makes the region of the plot (to the bottom and right) referring to the smaller scaffolds completely illegible. If this can be fixed, it might make the plot a little more interesting.

Are sufficient details of methods and materials provided to allow replication by others?

Yes

Is the rationale for creating the dataset(s) clearly described?

Yes

Are the datasets clearly presented in a useable and accessible format?

Yes

Are the protocols appropriate and is the work technically sound?

Yes

Reviewer Expertise:

(pan)genomics

I confirm that I have read this submission and believe that I have an appropriate level of expertise to confirm that it is of an acceptable scientific standard.
